# Specimen of a Well-Regulated Mad-House

**Published:** 1817-02

**Authors:** 


*For the London Medical and Physical Journal.*

### *Specimen of a well-regulated Mad-house;* by a Medical Observer.*

THE Legislature having commenced an enquiry respecting the state of the several provincial Lunatic Asylums, and having determined, that, by virtue of the bill now before Parliament, they shall, in future, be under the superintendance of government, and subject to certain regulations, agreeable to such Act of Parliament,—I think it but justice to say, that, when such enquiry shall be instituted, few Lunatic Asylums will be found more worthy the notice and patronage of the public, than the Asylum which has been long established at Droitwich, near Worcester.

Happening, some weeks past, to be at Droitwich, I was, by mere accident, induced to go with Mr. Ricketts through his well-arranged and extensive buildings. The good order and regularity which pervaded this asylum for unfortunate individuals labouring under one of the most deplorable maladies to which human nature is liable, made a forcible impression on my mind, and, at the same time, induced me to give this brief statement some degree of publicity, through the medium of your widely-extending Journal.

From a hasty conversation I had with Mr. Ricketts, I was much gratified to find, that, in many cases of incipient mania, he had, by his humane and skilful management, effected the most perfect and permanent recoveries. It was pleasing to remark the attachment and respect shewn by many of the unfortunate patients to Mr. Ricketts, as we passed through the different apartments of this asylum; as well as to observe the extreme cleanliness, warmth, and comfort they were enjoying, the rooms being, for the most part, heated by stoves, and frequently white-washed.—That portion of the building which is allotted to paupers is also in the most complete state of cleanliness and convenience,—far different from the wretched dwellings from which many of them had been taken, and which have been so justly described by the inimitable pen of Mr. Crabbe, in his poem of "the Parish Work-house," viz.

"Here, on a matted flock, with dust o'erspread,

The drooping wretch reclines his languid head,

* Though this article appears anonymous, the writer has favoured us with his name, without which the paper would not have been inserted. We think it right to add, that, in a number of visits we have made to Bethlem, when under the care of different officers, we never saw this mixture of the two sexes; and strongly suspect our correspondent's zeal has been greater than his memory.—Edit.

Dejected widows with unheeded tears,
And crippled age, with more than childhood fears;
The lame, the blind, and far the happiest they,
The moping idiot and the madman gay."

The cells, in which are placed such maniacs as require close confinement, are remarkably well ventilated, and are so contrived as to admit a stream of water to be passed through them, whenever it is deemed necessary.

Amongst the upper order of lunatics, whose cases do not require strict confinement, a very proper regard is paid to their former rank and habits of life; and every kind indulgence is allowed and provided, that is not esteemed incompatible with the medical treatment of the particular species of mania under which they labour.

It is now upwards of twenty-five years since I accompanied the late Mr. John Hunter to Bethlem Hospital, and where I could not help being struck with astonishment at the heterogeneous mixture of maniacs which, at that time, presented themselves in this immense building, where, except such as were in a state of raving madness, men and women were allowed to be together in a promiscuous manner, walking about apparently without any person to superintend them; some who appeared to be in a state of confirmed mania, and others in more incipient states of derangement. It is presumed "they manage these things better" in the present period of investigation and improvement. The contrast, however, by recollection, appeared singularly striking, when compared with the Asylum of Mr. Ricketts, where the inmates were so judiciously classed, and so regularly attended to in every respect.

I have withheld my name from this brief statement of facts, lest I might be supposed to be induced by some sinister or partial motive, by which I should assuredly not be influenced on so important a subject, having merely given this account in testimony of the scene I casually witnessed, and in the hope that the good order and discipline of this Asylum may be more generally known.

*Dec.* 17, 1816.

